# Integrative genomic analysis of drug resistance in *MET* exon 14 skipping lung cancer using patient-derived xenograft models

**DOI:** 10.3389/fonc.2022.1024818

**Published:** 2022-10-21

**Authors:** Yunhua Xu, Linping Gu, Yingqi Li, Ruiying Zhao, Hong Jian, Wenhui Xie, Liu Liu, Huiwen Wu, Fang Ren, Yuchen Han, Shun Lu

**Affiliations:** ^1^ Department of Shanghai Lung Cancer Center, Shanghai Chest Hospital, School of Medicine, Shanghai Jiao Tong University, Shanghai, China; ^2^ GenomiCare Biotechnology (Shanghai) Co., Ltd., Shanghai, China; ^3^ Department of Pathology, Shanghai Chest Hospital, School of Medicine, Shanghai Jiao Tong University, Shanghai, China; ^4^ Department of Nuclear Medicine, Shanghai Chest Hospital, School of Medicine, Shanghai Jiao Tong University, Shanghai, China; ^5^ Department of Nutrition, Shanghai Chest Hospital, School of Medicine, Shanghai Jiao Tong University, Shanghai, China; ^6^ EpimAb Biotherapeutics Co., Ltd., Shanghai, China

**Keywords:** TKI resistance, lung cancer, patient-derived xenograft, EGFR-MET bispecific antibody, MET exon 14 skippings

## Abstract

**Background:**

Non-small cell lung cancer (NSCLC) driven by *MET* exon 14 skipping (*MET*ex14) occurs in 3-4% of NSCLC cases and defines a subset of patients with distinct characteristics. While MET targeted therapy has led to strong clinical results in *MET*ex14 patients, acquired drug resistance seemed to be unavoidable during treatment. Limited information is available regarding acquired resistance during MET targeted therapy, nor has there been any report on such patient-derived xenografts (PDXs) model facilitating the research.

**Methods:**

We describe a patient case harboring *MET*ex14 who exhibited drug resistance after treatment with crizotinib. Subcutaneous xenografts were generated from pretreatment and post-resistance patient specimens. PDX mice were then treated with MET inhibitors (crizotinib and tepotinib) and EGFR-MET bispecific antibodies (EMB-01 and amivantamab) to evaluate their drug response *in vivo*. DNA and RNA sequencing analysis was performed on patient tumor specimens and matching xenografts.

**Results:**

PDXs preserved most of the histological and molecular profiles of the parental tumors. Drug resistance to MET targeted therapy was confirmed in PDX models through *in vivo* drug analysis. Newly acquired *MET* D1228H mutations and *EGFR* amplificated were detected in patient-resistant tumor specimens. Although the mutations were not detected in the PDX, EGFR overexpression was observed in RNA sequencing analysis indicating possible off-target resistance through the EGFR bypass signaling pathway. As expected, EGFR-MET bispecific antibodies overcome drug resistant in the PDX model.

**Conclusions:**

We detected a novel MET splice site deletion mutation that could lead to *MET*ex14. We also established and characterized a pair of *MET*ex14 NSCLC PDXs, including the first crizotinib resistant *MET*ex14 PDX. And dual inhibition of MET and EGFR might be a therapeutic strategy for EGFR-driven drug resistance *MET*ex14 lung cancer.

## Introduction

Non-small cell lung cancer (NSCLC) is a heterogeneous disease that accounts for 85% of lung cancer diagnoses, accompanied by various alterations in known oncogenes including *EGFR*, *ALK*, *ROS* and *MET*. *MET* exon 14 skipping (*MET*ex14), often reported without co-occurring oncogenic driver mutations, has been identified in 3-4% of NSCLC cases and are associated with poor prognosis (1, 2).

*MET* is a proto-oncogene that encodes for a tyrosine kinase receptor and plays key functions in embryonic development, organogenesis and wound healing. Somatic mutation in introns 13 or 14 of *MET* could lead to an alternative splicing result in the skipping of exon 14 (3). Exon 14 encodes for the cytoplasmic juxtamembrane domain responsible for ubiquitination of MET protein, which maintains the dynamic balance between MET activation and its removal from the cell surface. A lack of the juxtamembrane domain disrupts the regulation of MET signaling causing persistent activation of the downstream signaling pathway (4, 5).

Considering that *MET*ex14 tends to occur in the absence of other oncogenic mutations, MET activity is the key event in tumorigenesis. Clinical evidence suggests that MET specific tyrosine kinase inhibitors (TKIs) are highly effective against tumors bearing *MET*ex14. Capmatinib and tepotinib have recently been approved for the treatment of NSCLC patients harboring *MET*ex14, and National Comprehensive Cancer Network (NCCN) guidelines also note that crizotinib may be useful in certain circumstances and is recommended as a category 2A treatment option (6–9). In addition, several other MET inhibitors have already shown clinical benefits for *MET*ex14 NSCLCs, including savolitinib, merestinib and bozitinib (10).

Despite the dramatic initial response to MET inhibitor therapy, patients eventually develop drug resistance through activation of the bypass signaling pathway (off-target) or MET resistant mutations (on-target) (11). This has limited the potential impact of this form of therapy. Thus, identification of the precise drug resistance mechanism(s) affecting MET inhibitors will be essential to enable clinicians to accommodate therapeutic strategies in pursuance of improved clinical outcomes. Preclinical models such as a patient-derived xenograft (PDX) model have become increasingly important in translational research. The reason is that PDX models are believed truly to resemble the original disease, therefore providing researchers with invaluable insights.

Previously, our group has successfully generated an ALK+ and EGFR+ targeted TKI resistance PDX model using novel microfluidic technology to investigate underlying resistance mechanisms (12). In the present study, we established two PDX models from a *MET*ex14 NSCLC patient’s tumor specimen that were taken before treatment and after resistance to crizotinib developed. To date and as far as we are aware, there have been no published articles on PDX models representing TKI-resistant *MET*ex14 patients. Thus, we aim to improve the understanding of the underlying mechanisms involved in TKI drug resistance.

## Patient and methods

### Patient specimens

Primary tumor specimens at diagnosis and resistance specimens after crizotinib treatment were obtained using CT-guided biopsy. Tumor specimens and their paired peripheral blood samples were collected to establish PDX and to conduct DNA whole exome-sequencing (WES) and RNA sequencing (RNA-seq).

### Animals

Female NOD SCID mice were purchased from the Beijing Vital River Laboratory Animal Technology Co., Ltd and were between 6-8 weeks of age at the time of implantation. Mice were hosted in a specific pathogen-free (SPF) environment of a vivarium facility and acclimatized to their new environment for at least three days before the initiation of any experiments following IACUC protocols.

### Establishment of xenograft models

Tumor biopsy specimens from the patient were implanted subcutaneously in the right flanks of female NOD SCID mice using an 18-gauge trocar needle. The first implanted passage was defined as P0 mice (tumor), the next passage implanted from P0 tumor was defined as P1 mice (tumor), and further passages were generated by serial implantation. Tumor sizes were measured routinely in two dimensions using a caliper, and the volume was expressed in mm^3^ using the formula: V = 0.5a x b^2^, where a and b are the long and short diameters of the tumor, respectively.

### Immunohistochemistry

Tissue samples were collected, formalin-fixed and paraffin-embedded (FFPE) using Leica Peloris II Tissue Processor (Leica Biosystems, Germany). Then, slides were Hematoxylin and Eosin (H&E) stained by HistoCore SPECTRA ST Stainer (Leica Biosystems, Germany) and were assessed by two experienced pathologists. IHC staining was performed on BenchMark ULTRA automated slide stainer (Roche Diagnostics, USA). The results of the immunostaining were scored according to the WHO classification of lung tumors (5th edition): positive staining on ≥1% tumor cells was defined as positive, otherwise defined as negative.

### *In vivo* drug treatments and efficacy evaluation

30 mm^3^ tumor fragment was implanted subcutaneously in the right flank of each mouse. When tumor reached an average volume of 100-150 mm^3^, the mice were randomly assigned to either control or treatment groups (n=5-6 mice/group). Mice were dosed orally daily with vehicle control, crizotinib at 50 mg/kg and tepotinib at 10 mg/kg. Amivantamab was injected intraperitoneally twice a week at 16 mg/kg, and EMB-01 was injected intraperitoneally once a week at 16 mg/kg. All drugs were administered following a strict schedule.

To evaluate the anti-tumor efficacy of each drug, the percentage of tumor growth inhibition (TGI) values was calculated with the formula:


TGI (%) = [1−(change of tumor volume in treatment group)/(change of tumor volume in vehicle group)] × 100

### Specimen extraction and sequencing

WES and various analyses were performed at the Genomics Laboratory of GenomiCare Biotechnology (Shanghai, China). Specimen extraction and sequencing were performed following a previously published protocol (13–15). DNA in frozen tissue specimens and blood were extracted from thawed materials with the Maxwell RSC Blood DNA Kit (cat# AS1400, Promega, Madison, WI, USA) on a Maxwell RSC system (cat# AS4500, Promega). DNA of FFPE tissue was extracted using the MagMAX FFPE DNA/RNA Ultra Kit (cat# A31881, ThermoFisher, Waltham, MA, USA) on a KingFisher Flex system (ThermoFisher). The extracted DNA was sheared by a Covaris L220 sonicator and the exome DNA was captured using the SureSelect Human All Exon V7 kit (cat# 5991-9039EN, Agilent). The SureSelectXT Low Input Target Enrichment and Library Preparation system (cat# G9703-90000, Agilent, Santa Clara, CA, USA) was used for target enrichment and library preparation. DNA aliquots were then sequenced on an Illumina NovaSeq-6000 sequencer (Illumina, San Diego, CA, USA) to generate 2 x 150 bp paired-end reads. Image analysis and base calling were conducted using onboard RTA3 software (Illumina) (13, 14).

RNA from FFPE specimens was purified using the MagMAX FFPE DNA/RNA Ultra Kit (cat# A31881, ThermoFisher) on a KingFisher Flex system (ThermoFisher) and used as the template to synthesize cDNA using the NEBNext RNA First Strand Synthesis Module (cat# E7525S, NEB, Waltham, MA, USA) and the NEBNext mRNA Second Strand Synthesis Module (cat# E6111S, NEB) sequentially. The library preparation, sequencing and base calling were conducted similarly as described above in the WES section) (15).

### Somatic variant identification

After removing adapters and low-quality reads, the commercial Sentieon (version 201911) (16) running environment with default parameters was implemented to process the following steps sequentially: reads alignment to NCBI human genome reference assembly hg19 using the Burrows‐Wheeler Aligner (BWA) algorithm, duplication sorting, realignment and recalibration, and somatic mutation calling including single nucleotide variations (SNVs) and short insertion/deletions (INDELs). During the mutation calling stage, the reads from the tumor specimens were compared with paired blood from the same patient to generate the somatic mutation list. The called somatic mutations were then filtered to retain only the mutations with the variant allele frequency (VAF) ≥ 0.05 and supported by at least three reads, and annotated using the Variant Effect Predictor (VEP) package (13, 14).

### Copy number variation

By following the ExomeCNV package (17), a normalized depth-of-coverage ratio approach was used to identify CNV from the WES results of paired specimens. A standard normal distribution was used to account for five sources of bias that would affect raw read counts, which included the size of exonic regions, batch effect, the quantity and quality of the sequencing data, local GC content and genomic mappability. Genes with a haploid copy number ≥ 3 or ≤ 1.2 were defined as amplified or deleted, respectively and a minimum tumor content (purity) of 20% was required (13, 14).

### RNA differential gene expression analysis

RNA-seq reads were mapped to the NCBI human genome reference assembly hg19 using STAR (version 020201) (18) and generated a count matrix. The raw read counts were further normalized by log2-counts per million normalization (TPM) (15).

### Statistical analysis

Data were presented as mean ± standard error. Tumor growth curves were analyzed using two-way analysis of variance (ANOVA) with Bonferroni’s correction by GraphPad Prism 7.0 software (GraphPad Inc, San Diego, CA, USA).

## Results

### Patient case

**Figure 1:**
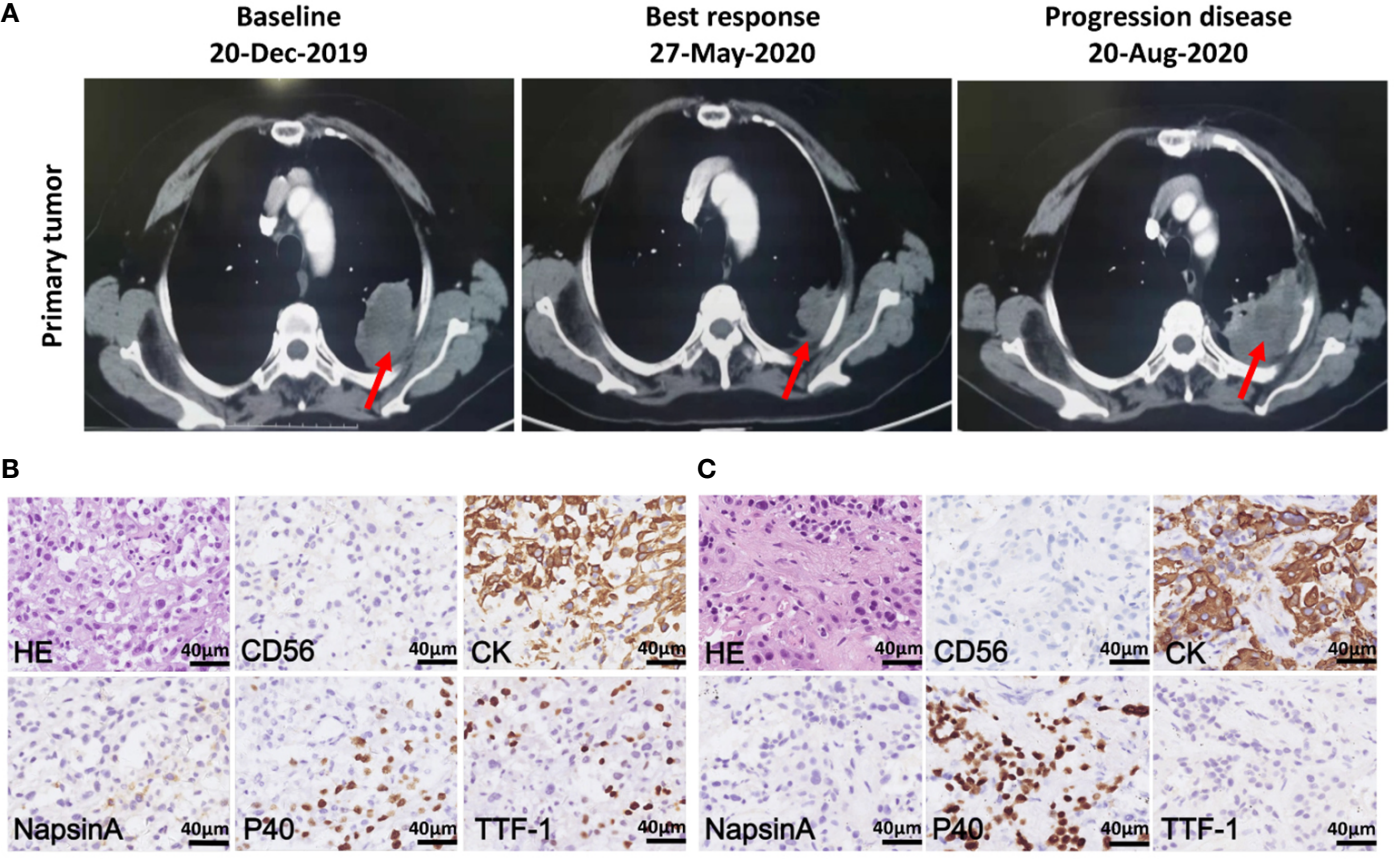
Chest computed tomography (CT) scans and histopathology of patient tumor before and after crizotinib treatment. **(A)** CT scans of the primary tumor of patient at baseline (left), best response (middle) and progression disease (right) after crizotinib treatment. Red arrows point to malignant loci. **(B)** H&E staining and IHC staining of the tumor biopsies from patient before crizotinib treatment, which was CD56 negative, CK positive and partially positive for TTF1, P40 and NapsinA; the scale bar represents 40 μm. **(C)** H&E staining and IHC staining of tumor biopsy specimens from a patient in disease progression, which was negative for CD56, NapsinA and TTF-1, positive for CK and P40; the scale bar represents 40 μm.

In December, 2019, a 68-year-old non-smoker female patient with a history of cavernous sinus hemangioma surgery was hospitalized at the Shanghai Chest Hospital. Chest computed tomography (CT) scan revealed a 5.2 x 4.5 x 6.2 cm mass in the left upper lobe with multiple metastatic sites at mediastinal lymph nodes, bone, and brain (**Figure 1A**). Bioposy at upper left lung nodule was performed for pathological analysis, next-generation sequencing (NGS), and the establishment of a xenograft. The patient was diagnosed with stage IV non-small cell lung cancer-not otherwise specified (NSCLC-NOS) harboring *MET* exon 14 mutation (**Figure 1B**). Patient received crizotinib treatment starting January 2020, and achieved a partial response where the tumor size reduced by 35.5%. Patient continued to recieve crizotinib for 7 months until diseased progressed (**Figure 1A**). At time of disease progression, the patient again underwent a CT-guided biopsy of the left upper lung lesion for pathology tests, NGS, and the establishment of a xenograft. Histopathological examination revealed squamous cell carcinoma characteristics this time (**Figure 1C**). Due to poor physical condition and ECOG score, patient received best supportive care for 4 months until she passed away in December 2020.

### Establishment of patient-derived xenograft models for human *MET*ex14 NSCLC with and without crizotinib resistance

**Figure 2:**
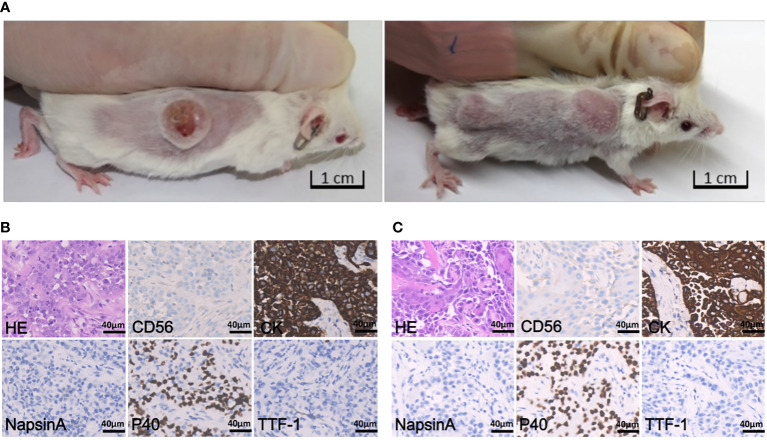
Tumor-bearing mice and histopathology staining. **(A)** Representative images of tumor-bearing PDX mice established from patient pretreatment tumor (left) and drug-resistant tumor (right), respectively. **(B)** H&E staining and IHC staining of tumor derived from pretreatment PDX (passage 3), which was negative for CD56, NapsinA and TTF-1, and positive for CK and P40; the scale bar represents 40 μm. **(C)** H&E and IHC staining of tumor derived from drug- resistant PDX (passage 2), which is negative for CD56, NapsinA and TTF-1, and positive for CK and P40; the scale bar is 40 μm.

The subcutaneous models were established from biopsy specimens obtained before (PDX-pretreatment) or after (PDX-resistant) crizotinib resistance had developed. PDXs were serially passaged in animals 3-5 times for tissue expansion. Representative tumor-bearing mice are shown in **Figure 2A**. No significant body mass loss was observed in mice bearing both PDX tumors.

The histology and degree of differentiation of the PDX-pretreatment tumor was slightly different from the original tumor. Patient was initially diagnosed with NSCLC-NOS, whereas PDX sample was diagnosed with squamous cell carcinoma (**Figure 2B**). Histology of PDX-resistant tissue matched well with its original tumor (**Figure 2C**). In addition, both PDX tumors maintained intra-tumor heterogeneity, resembling the original tumors.

**Figure 3:**
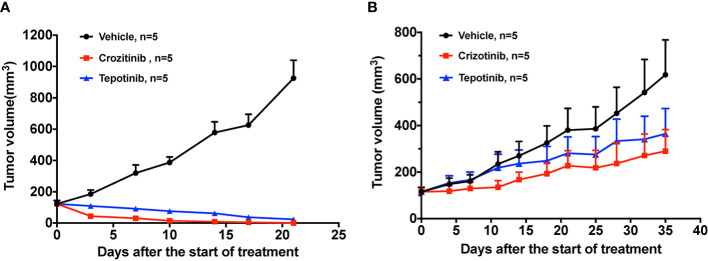
The effect of TKIs on two PDX models. **(A)** Tumor change in pretreatment PDX mice treated with vehicle, 50 mg/kg crizotinib and 10 mg/kg tepotinib once a day for 3 weeks. **(B)** Tumor change in drug resistant PDX mice treated with vehicle, 50 mg/kg crizotinib and 10 mg/kg tepotinib once a day for 5 weeks. Tumor volume was measured and expressed as the mean ± standard error.

To evaluate the responses of PDX models to the standard-of-care agent, we conducted *in vivo* efficacy studies of crizotinib and tepotinib in two PDX models. Crizotinib and tepotinib administration all proved to be highly effective in suppressing PDX-pretreatment tumor growth. The quantified TGI at day 21 were 115.25% and 112.31%, respectively (**Figure 3A**). While drug resistant PDXs showed decreased drug sensitivity at day 35 to crizotinib (TGI = 65.32%) and tepotinib (TGI = 50.40%), they resembled the patient’s drug resistance to crizotinib (**Figure 3B**).

### Sequencing data validated the PDX model and reported on aquired target *MET* mutation and *EGFR* gene amplification in a patient crizotinib-resistant specimen

**Figure 4:**
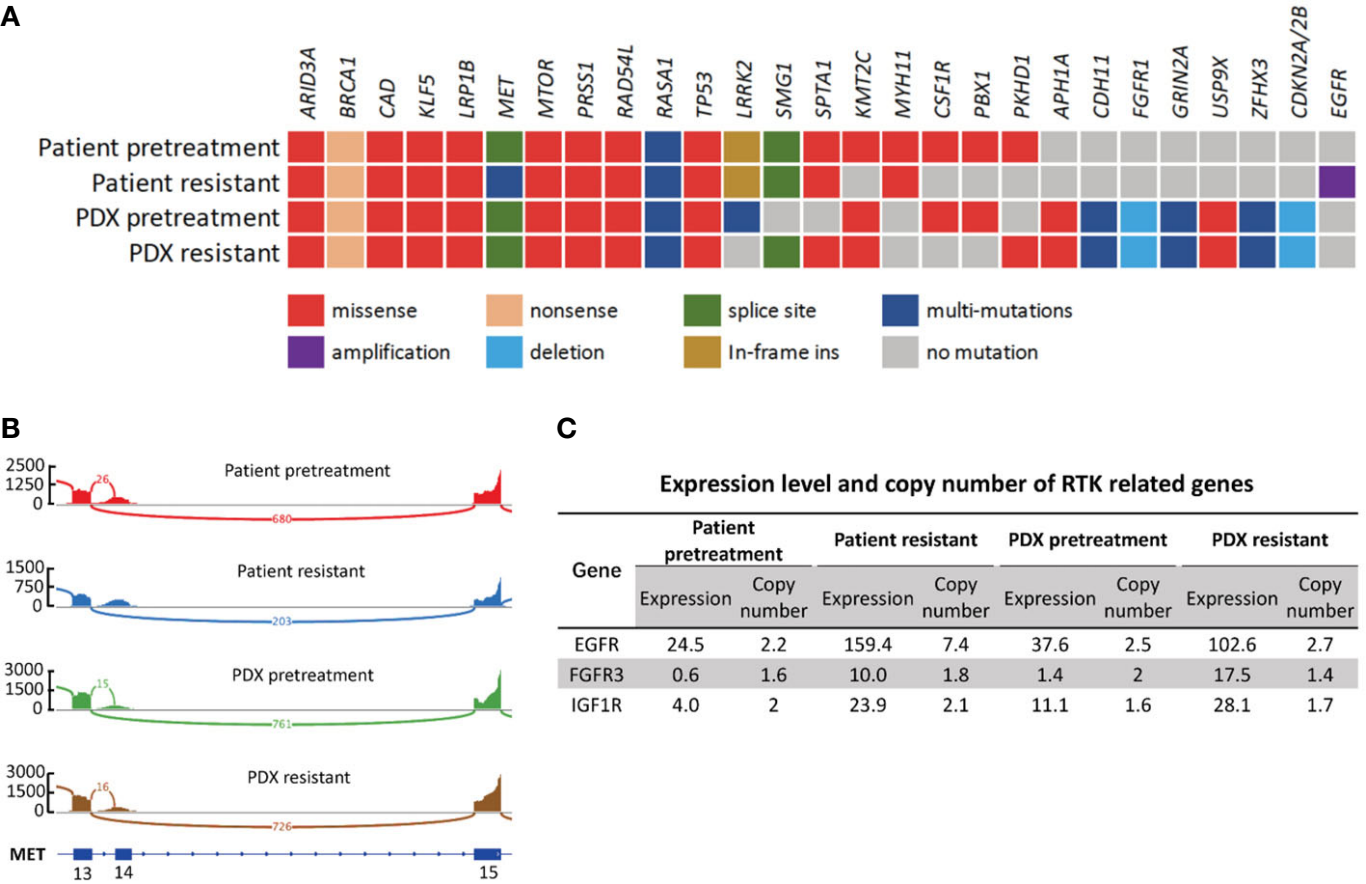
Genetic and transcriptomic characterization of tumors from the patient and the corresponding PDX models. **(A)** Co-mutation plot of genomic alterations across tumor specimens from patient and PDX models. Mutations and frequency of each gene are listed in **Supplementary Table S1**. **(B)** Sashimi plot of *MET* exon14 skipping event in patient and PDX tumor specimens. The Y axis indicates read density, the number on arc represents junction reads, and portion of schematic *MET* transcripts with exon number is the plot at the bottom. The figure was adapted from IGV (19). **(C)** Expression level and copy number of RTK related genes in patient and PDX model tumor specimens. Numbers are presented in TPM.

WES and RNA-seq were performed on patient biopsy specimens before treatment and after resistance to crizotinib developed and on their corresponding PDX tissue. WES and RNA-seq results confirmed that both PDX models retained *MET*ex14 as original tumors (**Figures 4A, B**). DNA results revealed that both PDX tumors retained the overall pattern of the somatic mutations and copy number variation of their original tumor tissue. **Figure 4A** gives a list of a total of 27 mutations, either oncogenic or with higher relevance to the disease. Most of the oncogene alterations in the parent primary tumor were preserved in the PDX specimens including *MET*ex14, *MTORI* ^2500F^, *RAD54L^G235R^*, *BRCA1* ^Q1240*^ and *TP53* ^H193R^ (**Figure 4A**). We noticed the frequency of several mutations, including *MET*ex14 (splice site p.E1009fs), *TP53* ^H193R^, *ARID3A* ^P373L^ and *PRSS1* ^T137M^, in PDX tissue increased to 100% which may be a result of a homozygous mutation in the original tumor.

We then compared resistant specimens to pretreatment specimens in patient and PDX models. *MTOR* ^I2500F^, *RAD54L* ^G235R^, *BRCA1* ^Q1240*^, *TP53* ^H193R^ and *MET*ex14 (splice site p.E1009fs) were fairly consistent between the 2 patient specimens, while mutations of *KMT2C* ^G4660E^, *FGFR1* ^N546K^, *PBX1* ^E60K^ and *CSF1R* ^L368V^ were lost in patient-resistant specimens. *MET* ^D1228H^ and *APC* ^D396N^ newly occurred in patient-resistant specimens at mutational frequencies of 6.44% and 5.08%, respectively (**Supplementary Table S1**). However, these two mutations were lost in PDX-resistant tissue at both the DNA and the RNA level, possibly implying clonal selection during establishment.

**Figure 5:**
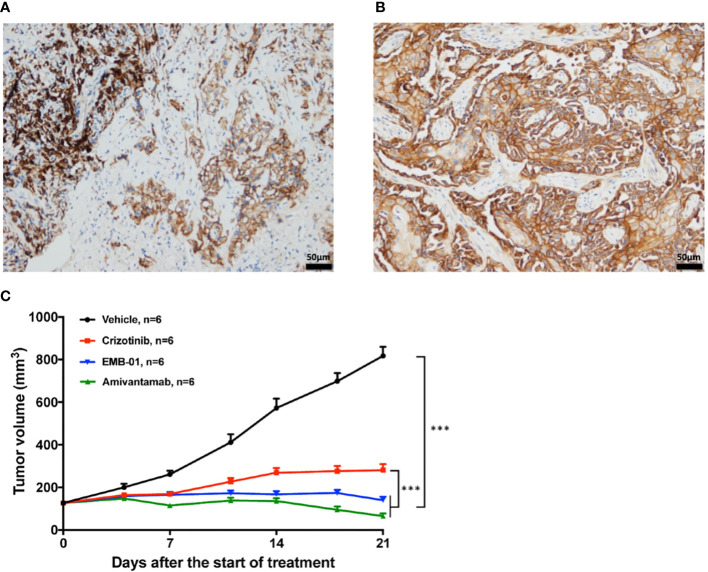
Representative pictures for EGFR IHC staining and the effect of EGFR/cMET bispecific antibody on drug resistant PDX model. **(A)** IHC staining of EGFR in patient-resistant sample. **(B)** IHC staining of EGFR in PDX-resistant sample. **(C)** Tumor change in drug resistant PDX mice treated with vehicle, 50mg/kg crizotinib once daily, 16mg/kg EMB-01 once a week, 16mg/kg Amivantamab twice a week for 3 weeks. Tumor volume was measured and expressed as the mean ± standard error. ***p< 0.001 by two-way ANOVA Bonferroni’s correction.

*EGFR* amplification, a classic drug resistance gene alteration, was detected in patient-resistant specimens where the copy number of *EGFR* significantly increased from 2.24 to 7.44 (**Figure 4C**). This gene amplification was not found in PDX-resistant tissue. However, we did observe a significant increase in the RNA expression level of *EGFR* in PDX-resistant tissue compared to pretreatment tissue. Expression value increased from 37.6 to 102.6, which was consistent with patient specimens. EGFR overexpression was confirmed by IHC staining in both patient-resistant and PDX-resistant tissues. Result showed strong and diffuse membranous and cytoplasmic staining in both PDX and patient resistant tissues (**Figures 5A, B**). Since *EGFR*, *FGFR3*, *IGF1R* and *MET* all belong to the RTK family, we further investigated the RNA expression levels in these RTK-related molecules. RNA-seq data showed increased expression levels of *EGFR*, *FGFR3* and *IGF1R* in resistant specimens compared to pretreatment specimens indicating activation of the bypass signaling pathway (**Figure 4C**).

### Dual inhibition of MET and EGFR overcomes drug resistance in PDX model

To test whether EGFR overexpression contributes to drug resistance in mice model, we treated the drug-resistant PDX model with the EGFR-MET bispecific antibody EMB-01 (20) and amivantamab (21), which could inhibit both EGFR and MET signaling pathways. Results showed that both bispecific antibody treatment significantly inhibited tumor growth compared with crizotinib and vehicle treatment (**Figure 5C**). The quantified TGI at day 21 for crizotinib, EMB-01 and amivantamab were 77.5%, 98.1% and 108.8%, respectively. Inhibition of crizotinib resistant tumor growth using EGFR-MET bispecific antibody affirmed our theory on EGFR overexpression as a bypass signaling pathway in this patient case.

## Discussion

In this study, we detected a novel *MET* c.3078_3082+6del alteration which could lead to splice of the *MET* exon 14. The alteration was initially annotated by bioinformatics software as a frameshift mutation (splice site p.E1009fs). Later this alteration was confirmed to be *MET*ex14 by RNA-seq. This patient received *MET* targeted therapy for harboring this alteration and showed partial response as described in the results section. It is critical for us to report this novel alteration to ensure patients with this mutation are provided the opportunity to receive the appropriate targeted therapy.

*MET* exon 14 skipping is known to be an important recurrent alteration that is responsible for driving tumorigenesis in NSCLC. Much research has proven that this oncogenic driver is mutually exclusive with other NSCLC driver mutations such as *EGFR*, *ERBB2*, *BRAF*, *MET* or *KRAS* (22). Cohort studies also reported distinct mutation patterns coincident with driver mutations suggesting distinct molecular subtypes in NSCLC (23). Common co-occurring mutations in *MET*ex14 patients included *TP53*, *NF1*, *BRAF* and *CDKN2A*; however, these co-alterations did not show a correlation to the development of MET inhibitor resistance (24). Resistance to TKI treatment can be mainly categorized into two types of mechanism, namely on-target and off-target resistance. On-target resistance usually occurs with a second-site mutation of the targeted oncogene or acquired oncogene gain or loss. Off-target resistance occurs through mutational activation of the downstream signaling pathway or the parallel bypass signaling pathway including the RAS-MAPK, PI3K-AKT and JAK-STAT pathways (25).

The *de novo* on-target second site *MET* mutations in the residue D1228 have been reported in several cases in response to crizotinib treatment (26). Protein crystallization of crizotinib with the kinase domain of MET suggested that a point mutation at this location could affect the binding affinity of crizotinib, due to the electrostatic change that resulted from the substitution (27). *In vitro* experiments also confirmed the loss of sensitivity towards crizotinib and other type I TKIs in cell lines harboring secondary *MET* ^D1228H^ mutation (28, 29). However, the PDX model of the resistant tumor in our study did not retain this mutation. The low allelic fraction of this *de novo* mutation in NGS results indicated that clonal selection, sites of biopsy, and clonal evolution during tumor establishment and passaging were likely the cause for the discrepancy (30, 31). This loss of the resistant mutation led us to propose the involvement of other contributing factors in the process of gaining drug resistance.

Acquired *EGFR* gene amplification in patient-resistant tissue suggested that activation of the EGFR related pathway could be another contributor to crizotinib resistance in this case. *EGFR* amplification has been found in *MET*ex14 patients as a resistance mechanism during MET targeted therapies (26). Acquired *MET* amplification has been extensively described in the literature, stressing the biological link between EGFR and MET (32, 33). *In vivo* experiments on a MET-resistant gastric cancer cell line revealed that EGFR signaling became the primary and independent driver for the downstream signaling pathway mitogen-activated protein kinase (MAPK)/extracellular signal-related kinase (ERK) (21). MET activation also signals through the MAPK/ERK pathway regulating cell proliferation, cell motility and cell cycle progression (34). Combined inhibition of EGFR and MET that successfully overcame the resistance towards MET targeted TKI resistance has been described *in vitro* and *in vivo* (26, 35). These results suggested that MET and EGFR are functionally redundant in maintaining the activation of the downstream signaling pathway in targeted TKI inhibitor resistance (36).

As described in the results section, our drug resistant PDX model retained *EGFR* overexpression from the original tumor but not *EGFR* gene amplification. Although gene amplification is a strong indicator of EGFR overexpression, inconsistency between *EGFR* amplification and overexpression has been observed in many cases, showing *EGFR* amplification is sufficient but not necessary for transcriptome level overexpression (37). Our IHC results also confirmed EGFR overexpression in resistant PDX model supporting our theory.

Successfully inhibited tumor growth using EGFR-MET bispecific antibody in drug resistant PDX model supported our theory regarding the role of EGFR activation in the resistance to MET-targeted TKIs. By using the first crizotinib resistant *MET*ex14 NSCLC PDX, our result provided evidence *in vivo* to support using bispecific antibody as a clinical strategy to overcome EGFR-driven MET TKIs resistant NSCLC (38–41).

## Data availability statement

The datasets presented in this study can be found in online repositories. The names of the repository/repositories and accession number(s) can be found below: BioProject accession code PRJNA765468.

## Ethics statement

The studies involving human participants were reviewed and approved by Shanghai Chest Hospital Research Ethics Committee at Shanghai Chest Hospital. The patients/participants provided their written informed consent to participate in this study. The animal study was reviewed and approved by Shanghai Chest Hospital Research Ethics Committee at Shanghai Chest Hospital. Written informed consent was obtained from the individual(s) for the publication of any potentially identifiable images or data included in this article.

## Author contributions

YHX, LPG, HJ, and SL conceived this study. YHX, LPG, YQL, RYZ, HJ, WHX, LL and HWW collected data. YHX, LPG, YQL, RYZ, HJ, YCH, WHX, LL, FR and SL performed data analysis and interpretation. YHX, LPG, YQL, RYZ and YCH drafted the manuscript. YHX, LPG, YQL, RYZ, YCH and SL revised and commented on the draft. All authors contributed to the article and approved the submitted version.

## Fundings

This work was supported by Science and Technology Commission of Shanghai Municipality Project (grant no. 19140902600), Shanghai Chest Hospital Project of Collaborative Innovation Grant (grant no. YJXT20190210), Shanghai Chest Hospital Project (grant no. 2019YNJCM06), the Technology Transfer Project of Shanghai Jiao Tong University School of Medicine (No. ZT202010) and Clinical Research Plan of SHDC (grant no. 16CR3102B).

## Acknowledgments

The authors would like to thank Guan Wang and his colleagues at GenomiCare Biotechnology (Shanghai) Co., Ltd., for technology supports and discussion.

## Conflict of interest

Author YQL was employed by the company GenomiCare Biotechnology (Shanghai) Co, Ltd. Author FR was employed by EpimAb Biotherapeutics Co., Ltd. 

The remaining authors declare that the research was conducted in the absence of any commercial or financial relationships that could be construed as a potential conflict of interest.

## Publisher’s note

All claims expressed in this article are solely those of the authors and do not necessarily represent those of their affiliated organizations, or those of the publisher, the editors and the reviewers. Any product that may be evaluated in this article, or claim that may be made by its manufacturer, is not guaranteed or endorsed by the publisher.

## Glossary

**Table:**

NSCLC	non-small-cell lung carcinoma
EGFR	epidermal growth factor receptor
ALK	anaplastic lymphoma kinase
ROS1	c-ros proto-oncogene 1
METex14	MET exon 14 skipping
MTOR	Mechanistic target of rapamycin kinase
RAD54L	RAD54 like
BRCA1	Breast cancer type 1
TP53	Tumor protein p53
KMT2C	Lysine Methyltransferase 2C
FGFR	fibroblast growth factor receptor
IGF1R	Insulin-like growth factor 1 receptor
PBX1	Pre-B-cell leukemia transcription factor 1
APC	Adenomatous polyposis coli
CSF1R	Colony Stimulating Factor 1 Receptor
ERBB2	Erb-B2 Receptor Tyrosine Kinase 2
BRAF	v-Raf murine sarcoma viral oncogene homolog B
KRAS	Kirsten rat sarcoma
NF1	Neurofibromatosis type 1
CDKN2A	Cyclin Dependent Kinase Inhibitor 2A
Ras-MAPK	Rat sarcoma virus GTPase/mitogen-activated protein kinase;
PI3K-AKT	phosphatidylinositol 3−kinase/protein kinase B
JAK-STAT	Janus kinase/signal transducer and activator of transcription
ERK	extracellular signal-regulated kinases
TPM	transcripts per million
RTK	receptor tyrosine kinases
TKI	tyrosine kinase inhibitor
PDX	patient derived xenograft
NCCN	National Comprehensive Cancer Network
NGS	next generation sequencing
ECOG	Eastern Cooperative Oncology Group
NSCLC-NOS	non-small-cell lung carcinoma-not otherwise specific
FFPE	formalin-fixed and paraffin-embedded
IACUC	The Institutional Animal Care and Use Committee
WHO	World Health Organization
SPF	specific pathogen-free
WES	whole exome-sequencing
IHC	immunohistochemistry;
DNA	deoxyribonucleic acid
RNA	ribonucleic acid
bp	base pair
cDNA	complementary DNA
CT	computed tomography
NOD SCID	nonobese diabetic/severe combined immunodeficiency
